# Modern Trends and Applications of Intelligent Methods in Biomedical Signal and Image Processing

**DOI:** 10.3390/s21030847

**Published:** 2021-01-27

**Authors:** Jan Kubicek, Marek Penhaker, Ondrej Krejcar, Ali Selamat

**Affiliations:** 1 Department of Cybernetics and Biomedical Engineering, VŠB-Technical University of Ostrava, 17. listopadu 15, 70 833 Ostrava-Poruba, Czech Republic; marek.penhaker@vsb.cz; 2 Center for Basic and Applied Research, Faculty of Informatics and Management, University of Hradec Kralove, Rokitanskeho 62, 50 003 Hradec Kralove, Czech Republic; ondrej.krejcar@uhk.cz; 3 Malaysia Japan International Institute of Technology (MJIIT), Universiti Teknologi Malaysia, Jalan Sultan Yahya Petra, Kuala Lumpur 54100, Malaysia; aselamat@utm.my

There are various modern systems for the measurement and consequent acquisition of valuable patient’s records in the form of medical signals and images, which are supposed to be processed to provide significant information about the state of biological tissues. Therefore, in the modern age of digital technologies in the healthcare sector, we are surrounded with big data of clinical patients containing valuable information about actual state and future prediction, which needs to be extracted from biomedical signals and images. Thus, the current trends in this area of biomedical engineering are focused on the design and development of intelligent methods, containing elements of artificial intelligence, allowing the extraction, classification, and optimization of clinical information from various medical data. Such methods significantly facilitate the workload of medical staff, and at the same time, serve to provide effective feedback for clinicians as decision-making systems. Particularly, such intelligent methods are employed for data smoothing, feature extraction, segmentation, identification, and classification. Such tasks require participation clinical specialists, mathematicians, and information experts, who together develop the intelligent systems which can be employed in the health care sector as a support to medical staff.

The Special Issue “Modern Trends and Applications of Intelligent Methods in Biomedical Signal and Image Processing” is aimed at the new proposals and intelligent solutions that constitute the state of the art of the intelligent methods for biomedical data processing from selected areas of signal and image processing. This Special Issue brings together research works from various fields that are related to the area of biomedical engineering to describe the recent trends and advances in this area.

For this Special Issue, we received 20 contributions in total. After judging scientific impact and novelty, we selected the 10 contributions included herein. The published papers include nine research papers and one review.

We appreciate all the authors who have decided to publish their research in this Special Issue. Thanks to these authors, we could provide the state of the art of the recent research of intelligent techniques in applications of biomedical engineering. Below, we summarize the individual contributions published in this Special Issue.

In recent years, image-guided navigation systems (IGNS) have become an important tool for various surgical operations. In the preparations for planning a surgical path, verifying the location of a lesion, etc., it is an essential tool; in operations such as bronchoscopy, which is the procedure for the inspection and retrieval of diagnostic samples for lung-related surgeries, it is even more so. In Reference [1], the authors propose a novel registration method to match real bronchoscopy images with virtual bronchoscope images from a 3D bronchial tree model built using computed tomography (CT) image stacks in order to obtain the current 3D position of the bronchoscope in the airways. This method represents a combination of a novel position-tracking method using the current frames from the bronchoscope and the verification of the position of the real bronchoscope image against an image extracted from the 3D model using an adaptive-network-based fuzzy inference system (ANFIS)-based image matching method. Experimental results show that the proposed method performs better than the other methods used in the comparison.

Heart problems are responsible for the majority of deaths worldwide. The use of intelligent techniques to assist in the identification of existing patterns in these diseases can facilitate treatments and decision making in the field of medicine. In Reference [2], authors extract knowledge from a dataset based on heart noise behaviors in order to determine whether heart murmur predilection exists or not in the analyzed patients. A heart murmur can be pathological due to defects in the heart, so the use of an evolving hybrid technique can assist in detecting this comorbidity team, and at the same time, extract knowledge through fuzzy linguistic rules, facilitating the understanding of the nature of the evaluated data. Heart disease detection tests were performed to compare the proposed hybrid model’s performance with the state of the art for the subject. The results obtained showed 90.75% accuracy, in addition to great assertiveness in detecting heart murmurs.

In recent years, research has focused on generating mechanisms to assess the levels of subjects’ cognitive workload when performing various activities that demand high concentration levels, such as driving a vehicle. These mechanisms have involved the implementation of several tools for analyzing the cognitive workload, and electroencephalographic (EEG) signals have been most frequently used due to their high precision. In Reference [3], the authors present a new feature selection model that is focused on pattern recognition using information from EEG signals based on machine learning techniques called GALoRIS (Genetic algorithms and logistic regression). This method utilizes genetic algorithms and logistic regression with the aim to make a new fitness function that identifies and selects the critical EEG features that contribute to recognizing high and low cognitive workloads and structures.

In the area of medical data processing, wavelet transformation is frequently used for various applications, including data decomposition, smoothing, feature extraction, and image segmentation. One of the essential steps is the selection of suitable wavelet settings, including the mother wavelet and the decomposition level. Since wavelet transformation offers plenty of settings, it is usually a complicated task to select the most appropriate settings. In Reference [4], the authors propose a novel scheme that is able to simultaneously evaluate the effectivity of selected wavelet settings via the form of the spatial 2D maps. The authors also study the effect of dynamical noise influence within wavelet smoothing by using the volumetric mapping. The authors report of the testing of these techniques on both 1D EMG signals and 2D medical images from various imaging modalities.

In Reference [5], the authors present a novel approach intended for the periodical testing of the function evaluation of fetal heart rate monitors. The proposed simulator was designed to be compliant with the standard requirements for the accurate assessment and measurement of medical devices. The accuracy of the simulated signals was evaluated, and it was shown to be stable and reliable. The generated frequencies showed an error of about 0.5% with respect to the nominal one, while the accuracy of the test equipment was within ±3% of the test signal set frequency. The proposed device ensures easy and fast testing of fetal heart rate monitors. Hence, it provides an effective way to evaluate and test the correlation of commercial devices.

The invasive method of fetal electrocardiogram (fECG) monitoring is widely used, with electrodes directly attached to the fetal scalp. There are potential risks, such as infection, and thus it is usually carried out during labor when required. Recent advances in electronics and technologies have enabled fECG monitoring from the early stages of pregnancy through fECG extraction from the combined fetal/maternal ECG (f/mECG) signal recorded noninvasively in the abdominal area of the mother. In Reference [6], the authors propose an end-to-end deep learning model which is aimed at the detection of fetal QRS complexes. The proposed model also contains the residual network (resNet) architecture. This net is able to adopt a novel 1D octave convolution (OctConv), which is focused on multiple temporal frequency features. This fact predetermines the memory reduction and computational demands.

Time-of-flight (ToF) sensors are the source of various errors, including the multicamera interference artifact caused by the parallel scanning mode of the sensors. In Reference [7], the authors present a novel importance map, which is based on the median filtration algorithm with the aim of suppressing interference artifacts. The proposed method is based on the processing of multiple depth frames. This method uses the interference region and application of the interpolation. Performance of the algorithm was evaluated on a dataset consisting of the real-world objects with different textures and morphologies against popular filtering methods based on neural networks and statistics.

In Reference [8], the authors present a proposal of using electrodes for the continual measurement of the glucose concentration for the purpose of specifying further hemodynamic parameters. The proposal includes the design of the electronic measuring system, the construction of the electrodes themselves, and the functionality of the entire system, verified experimentally using various electrode materials. The proposed circuit works based on the microammeter measuring the size of the flowing electric current, and the electrochemical measurement method is used for specifying the glucose concentration. The electrode system is comprised of two electrodes embedded in a silicon tube. The authors present testing indicating that even if the Ag/AgCl electrode appears to be the most suitable, showing high stability, gold-plated electrodes showed stability throughout the measurement, similarly to Ag/AgCl electrodes, but did not achieve the same qualities in sensitivity and readability of the measured results.

The next study [9] proposes a novel multinetwork intelligent architecture, containing a multiscale convolutional neural network (MSCNN) with a fully connected graph convolution network (GCN), named MSCNN-GCN, for the detection of musculoskeletal abnormalities via musculoskeletal radiographs. The effectiveness of this model was verified by comparing the performance of radiologists and three popular CNN models (DenseNet169, CapsNet, and MSCNN) with three evaluation metrics (accuracy, F1 score, and kappa score) using the MURA dataset (a large dataset of bone X-rays).

The intake of microbially contaminated food poses severe health issues due to outbreaks of serious foodborne diseases. Therefore, there is a need for the precise detection and identification of pathogenic microbes and toxins in food to prevent these concerns. Thus, understanding the concept of biosensing has enabled researchers to develop nano-biosensors with different nanomaterials and composites to improve the sensitivity as well as the specificity of pathogen detection. In Reference [10], the authors publish a review that summarizes various sensing methods used in foodborne pathogen detection; in addition, the authors focus on the design, technical principles, and advances in sensing systems.
